# Epidemiology of HIV/AIDS infected persons in the western part of Romania in 2013

**DOI:** 10.1186/1471-2334-14-S4-P24

**Published:** 2014-05-29

**Authors:** Voichița Lăzureanu, Teodora Moisil², Virgil Musta, Maria Cerbu², Emilia Nicoară, Narcisa Nicolescu, Ruxandra Laza, Alexandru Crişan

**Affiliations:** 1 Victor Babeş University of Medicine and Pharmacy, Timişoara, Romania; 2 Dr. Victor Babeş Clinical Hospital of Infectious Diseases and Pneumology, Timişoara, Romania

## 

HIV transmission continues in Romania, despite decades of concerned efforts to inform the public about HIV and its consequences and reduce individual risk behaviors.

This is a retrospective study carried out in 2013 in the western part of Romania, in the counties Arad, Caraş-Severin, Hunedoara and Timiş.

In 2013 there are 846 patients with HIV/AIDS in active evidence, 86.4% in combined antiretroviral therapy (cART). Despite intense counseling, 30 (4.1%) patients interrupted willingly their antiretroviral therapy. There are 93 new infected persons in 2013, mostly men who have sex with men (MSM). 10 children were born in 2013 from HIV infected mothers, 1 was diagnosed while pregnant, 1 at delivery, 8 were known with HIV/AIDS prior to pregnancy. All 10 newborns received prophylaxis with antiretrovirals for 6 weeks. 11 (1.33%) patients were diagnosed with tuberculosis, 10 with pulmonary tuberculosis and 1 with tuberculous meningoencephalitis.

HIV/AIDS infection remains an important public health problem in the Western part of Romania.

